# District Nursing: III. The Labourer and Her Hire

**Published:** 1918-06-29

**Authors:** 


### THE MATRONS' AND SISTERS' DEPARTMENT.

### DISTRICT NURSING.

### III.—The Labourer and her Hire.

Like most other work district nursing may be considered from various points of view, and one at least is that of the well-being of the worker. It is pretty generally agreed that it is to the public interest that this branch of nursing should be made attractive to the best type of nurse, which simply means that it should be adequately paid, for the work itself is attractive enough to the right kind of woman. Till quite recently it has been one of very few channels open to public-spirited women, and they have accepted the hardships rather than be denied the work. But times are changing, and the whole field of the world's work is being thrown open to such women as are able to enter it. Inevitably, therefore, it will come about—it is already coming about—that the very women most wanted as the wise friends and servants of the poor and uneducated are finding other ways of congenial and useful work opening to them where the labourer is reckoned rather more worthy of her hire.

The standard rate of pay at present is £35-£40 resident and £90-£100 non-resident, with perhaps £4 a year uniform allowance. Some individual associations reach a higher figure, but they are a very small minority. One is quite often met with a curious theory that the district nurse has some mysterious secrets of cheap living. In reality the opposite is the case. She usually has to pay rather heavily for her lodgings or rest content with very bad ones, as she is a troublesome lodger, often unpunctual to meals, with wet coats and muddy boots; she wants space for her appliances and perhaps for her bicycle, and the door-bell goes frequently with her messages. Other heavy items are laundry and shoe-leather, and of course she needs food, fire, and light like the rest of the world, and there is the probability of an old age to consider. If she has the wee cottage that many so much prefer the difficulties are not lessened, for she must keep a maid of sorts, and a maid must be fed and paid. Nurses are often accused of being retrogressive and of taking little interest in public affairs, but it would be difficult to indulge a taste for the liberal pursuits on the above funds, a penny paper and a library subscription is the most it would run to; the actual purchase of a few books simply couldn't be fitted in. In point of fact, however, the district nurse seems often to have a little private income, and sometimes a little assistance in the way of a furnished cottage or rooms is thrown in with her pay; either circumstance eases the situation enough for toleration.

The figure at which to place a satisfactory minimum wage admits, of course, of difference of opinion. Most district nurses themselves suggest £150.

The question of raising the necessary funds is not absolutely easy. Some local associations are purely charitable, but the greater number of them are financed partly by honorary and partly by provident subscriptions. In a great many cases the provident basis could be revised and extended, in others the population is too absolutely poor or too small within a workable area to provide the funds.

There is some discussion lately of the possibility that in the near future provident district nursing may form part of the activities of a Health Ministry, but any such Ministry would scarcely be in a hurry to raise current rates of pay! Meanwhile it might be practicable, and it would certainly be an interesting experiment, to ask all collectors for district nursing associations to say frankly when they next go their rounds that it has been decided that the nurses are too badly underpaid and that an attempt is being made to raise salaries 25 per cent. Will the subscriber therefore consent to raise his (or her) subscription 25 per cent.? He (or she) is almost certain to have had one or more increases in pay during the last two years, and will probably be possessed of quite a lively sense of fair play. If this experiment were successful it would be a very gratifying proof of the appreciation of the nurse's work. Incidentally it would be tending to raise the standard of pay permanently, or at any rate till something more fundamental could be considered.

